# Supplementary material for: Host Species Affects Bacterial Evenness, but Not Diversity: Comparison of Fecal Bacteria of Cows and Goats Offered the Same Diet
Source: Animals (Basel). 2022 Aug 9;12(16):2011. doi: 10.3390/ani12162011 (PMC9404439; doi:10.3390/ani12162011)
Supplement: Supplementary file 1 [file animals-12-02011-s001.zip › animals-1742411-supplementary.pdf]

Supplementary Material

Host species affects bacterial evenness, but not diversity: comparison of fecal bacteria of cows and goats fed the same diet

Tiziana Maria Mahayri [1,3], Kateřina Olša Fliegerová [1*], Silvana Mattiello [2], Stefania Celozzi [2], Jakub Mrázek [1], Chahrazed Mekadim [1], Hana Sechovcová[1,4], Simona Kvasnová [1], Elie Atallah [3] and Giuseppe Moniello [3]

1 Laboratory of Anaerobic Microbiology, Institute of Animal Physiology and Genetics, Czech Academy of Sciences, 14220 Prague, Czech Republic
2 Department of Agricultural and Environmental Sciences—Production, Landscape, Agroenergy, University of Milan, Milan, Italy
3 Department of Veterinary Medicine, University of Sassari, 07100 Sassari, Italy
4 Czech University of Life Sciences in Prague, 165 00 Prague, Czech Republic
* Correspondence: fliegerova@iapg.cas.cz; Tel.: +420-267-090-504

Table S1. Animals' characteristics

Sample name	Animal species	Age	Lactating	Pregnant
GN 1	Goat	4 years	Yes	No
GN 4	Goat	6 years	Yes	No
GN 6	Goat	4 years	Yes	No
GN 7	Goat	3 years	No	Yes
GN 14	Goat	3 years	No	Yes
GN 15	Goat	3 years	No	Yes
GN 16	Goat	4 years	No	Yes
GN 17	Goat	5 years	No	Yes
GN 21	Goat	3 years	No	Yes
GN 22	Goat	7 years	No	Yes
GN 23	Goat	5 years	No	Yes
GN 24	Goat	7 years	No	Yes
GN 25	Goat	2 years	No	Yes
GN 27	Goat	3 years	No	Yes
GN 28	Goat	7 years	No	Yes
GN 29	Goat	4 years	No	Yes
GN 30	Goat	6 years	No	Yes
CN 1	Cow	3 years	Yes	Yes
CN 2	Cow	3 years	Yes	Yes
CN 6	Cow	7 years	Yes	Yes
CN 9	Cow	7 years	Yes	Yes
CN 10	Cow	3 years	Yes	Yes
CN 15	Cow	4 years	No	Yes
CN 16	Cow	5 years	No	Yes
CN 17	Cow	8 years	No	Yes
CN 19	Cow	7 years	No	Yes
CN 20	Cow	7 years	No	Yes
CN 21	Cow	7 years	No	Yes
CN 22	Cow	7 years	No	Yes
CN 23	Cow	7 years	No	Yes
CN 24	Cow	8 years	No	Yes
CN 26	Cow	5 years	No	Yes
CN 28	Cow	9 years	No	Yes

Table S2. Summary of alpha diversity indices of bacterial community in cows and goats (means ± SD).

Index	Cows	Goats	*p*-value
Chao 1	877.19 ± 708	688.54 ± 909	0.16
Pielou Evenness	0.934 ± 0.01	0.918 ± 0.02	0.017*
Shannon	8.286 ± 1.2	7.498 ± 1.3	0.11
Faith pd	60.82 ± 20	56.16 ± 23	0.13
ASV[1]	604 ± 372	422 ± 398	0.13

ASV[1], Amplicon sequence variant.

* Significant difference ($p < 0.05$)

Table S3. Relative abundance (means ± SD) of bacterial taxa at phylum, class, order and family levels in the feces of goats and cows.

Taxonomic levels	Goats	Cows
**Phylum**		
Firmicutes	75 ± 4.3%	74.9 ± 2.5%
Bacteroidetes	17.7 ± 2.5%	19.5 ± 1.9%
Spirochaetes	1.7 ± 0.6%	0.3 ± 0.2%
Proteobacteria	2.2 ± 0.6%	2.1 ± 0.6%
Actinobacteria	1 ± 0.5%	0.5 ± 0.3%
Tenericutes	0.5 ± 0.3%	0.9 ± 0.2%
Patescibacteria	0.5 ± 0.4%	0.5 ± 0.2%
Planctomycetes	0.2 ± 0.08%	0.1 ± 0.1%
Lentisphaerae	0.5 ± 0.2%	0.7 ± 0.3%
Epsilonbacteraeota	0.4 ± 0.6%	0.07 ± 0.1%
Elusimicrobia	0.03 ± 0.02%	0.05 ± 0.03%
Kiritimatiellaeota	0.02 ± 0.01%	0.06 ± 0.04%
Cyanobacteria	0.02 ± 0.01%	0.005 ± 0.003%
Fibrobacteres	0	0.0005 ± 0.03%
Fusobacteria	0.0002 ± 0.01%	0
**Class**		
Clostridia	74.2 ± 4.3%	71.3 ± 2.5%
Bacteroidia	17.7 ± 2.6%	19.5 ± 2%
Spirochaetes	1.7 ± 0.6%	0.5 ± 0.2%
Bacilli	0.2 ± 0.1%	3 ± 2 %
Deltaproteobacteria	0.9 ± 0.3%	0.7 ± 0.2%
**Order**		
Clostridiales	74.1 ± 4.3%	71.2 ± 2.5%
Bacteroidales	17.7 ± 2.6%	19.5 ± 1.9%
Spirochaetales	1.7 ± 0.6%	0.5 ± 0.2%
Bacillales	0.06 ± 0.02%	2.9 ± 2.2%

Desulfovibrionales	0.9 ±0.3%	0.7 ±0.2%
**Family**		
Ruminococcaceae	38.7 ±2.5%	39.5 ±2.8%
Christensenellaceae	13.7 ±2.8%	7.7 ±1%
Rikenellaceae	7.7 ±1.2%	6 ±0.7%
Peptostreptococcaceae	2.9 ±2.3%	5.7 ±1.9%
Lachnospiraceae	10.2 ±2.7%	9 ±1.1%
Family XIII	5.2 ±1.4%	4.7 ±0.8%
Spirochaetaceae	1.7 ±0.6%	0.5 ±0.2%
Planococcaceae	0.03 ±0.01%	2.2 ±2%
p-2534-18B5 gut group	0.7 ±0.7%	1.4 ±0.5%
Prevotellaceae	2.2 ±0.5%	3.6 ±0.9%
Desulfovibrionaceae	0.9 ±0.4%	0.7 ±0.2%
p-251-o5	1.1 ±0.6%	0.4 ±0.2%
Bacteroidales RF16 group	1.5 ±0.9%	3 ±0.5%
Unclassified family within order Bacteroidales	1.8 ±1.3%	2.5 ±0.5%
Clostridiaceae 1	0.5 ±0.5%	0.5 ±0.2%
F082	0.5 ±0.5%	0.3 ±0.08%
Unclassified family within order Izimaplasmatales	0.4 ±0.2%	0.8 ±0.2%
Clostridiales vadinBB60 group	1.7 ±0.6%	3.3 ±0.4%
Paludibacteraceae	0.4 ±0.3%	1 ±0.3%
Peptococcaceae	0.7 ±0.3%	0.5 ±0.2%
Acidaminococcaceae	0.6 ±0.2%	0.3 ±0.1%
Saccharimonadaceae	0.5 ±0.4%	0.5 ±0.2%
Bacteroidaceae	0.8 ±0.3%	0.6 ±0.2%
**Genus**		
Christensenellaceae R-7 group	13.5 ±2.8%	7.6 ±1%
Ruminococcaceae UCG-005	13 ±4.4%	10.4 ±3.2%
Ruminococcaceae UCG-010	8.7 ±2%	14.6 ±1.4%
Alistipes	3.8 ±1.2%	4.1 ±0.7%
Romboutsia	2.5 ±1.7%	3.6 ±1.5%
Rikenellaceae RC9 gut group	3.4 ±1%	1.2 ±0.3%
Eubacterium coprostanoligenes group	3 ±1.2%	2.3 ±0.6%
Unclassified genus within family Ruminococcaceae	3 ±1.7%	2.4 ±0.5%
Family XIII AD3011 group	2.4 ±0.9%	2 ±0.5%
Unclassified genus within family Lachnospiraceae	3.3 ±0.9%	2.6 ±0.3%
Unclassified genus within family Lachnospiraceae	2.1 ±1.5%	2 ±1%
Unclassified genus within family Ruminococcaceae	2.1 ±1%	2 ±0.7%
Ruminococcaceae UCG-013	1.5 ±0.7%	1.3 ±0.6%
Unclassified genus within family Bacteroidales RF16 group	1.3 ±0.8%	2.7 ±0.5%
Unclassified genus within family Clostridiales vadinBB60 group	1.4 ±0.5%	3 ±0.4%

Unclassified genus within family Paludibacteraceae	0.4 ±0.3%	1 ±0.3%
Ruminococcaceae UCG-014	1.4 ±0.8%	1.3 ±0.4%
Ruminococcaceae UCG-009	1 ±0.6%	1.2 ±0.6%
Mailhella	0.9 ±0.3%	0.7 ±0.2%
Treponema 2	1.7 ±0.6%	0.5 ±0.2%
Solibacillus	0.008 ±0.003%	1.5 ±1.7%
Prevotellaceae UCG-003	1 ±0.4%	1.5 ±0.5%
Ruminococcaceae UCG-002	1 ±0.9%	0.5 ±0.2%
Paeniclostridium	0.09 ±0.3%	1.3 ±0.4%
Unclassified genus within family p-2534-18B5	0.7 ±0.7%	1.5 ±0.5%
Prevotellaceae UCG-004	0.7 ±0.5%	1.2 ±0.2%
Unclassified genus within order Bacteroidales	0.4 ±0.3%	0.7 ±0.5%
Ruminococcaceae NK4A214 group	1.2 ±0.7%	0.7 ±0.3%
Mogibacterium	1 ±1.1%	0.7 ±0.3%
Unclassified genus within order Bacteroidales	1 ±0.5%	0.2 ±0.1%
Ruminiclostridium 9	0.4 ±0.4%	0.7 ±0.7%
Unclassified genus within family Peptostreptococcaceae	0.3 ±0.6%	0.8 ±0.5%
Eubacterium brachy group	0.4 ±0.3%	0.5 ±0.3%
Clostridium sensu stricto 1	0.5 ±0.5%	0.5 ±0.1%
Escherichia-Shigella	0.5 ±0.2%	0.05 ±0.05%
Lysinibacillus	0.01 ±0.005%	0.7 ±0.5%
Unclassified genus within order Izimaplasmatales	0.4 ±0.2%	0.8 ±0.2%
Ruminococcus 1	0.6 ±1.3%	0.4 ±0.2%
Unclassified genus within family Peptococcaceae	0.6 ±0.3%	0.4 ±0.2%
Unclassified genus within family Family XIII	0.5 ±0.6%	0.5 ±0.4%
Unclassified genus within order Bacteroidales	1.3 ±1.4%	1.2 ±0.2%
Eubacterium nodatum group	0.5 ±0.6%	0.7 ±0.3%
Lachnospiraceae NK3A20 group	0.8 ±1.3%	0.6 ±0.3%
Lachnospiraceae AC2044 group	0.5 ±0.7%	0.2 ±0.1%
Unclassified genus within order Bacteroidales	0.009 ±0.06%	0.5 ±0.2%
Dorea	0.6 ±0.6%	0.5 ±0.3%
Phascolarctobacterium	0.6 ±0.2%	0.3 ±0.1%
dgA-11 gut group	0.5 ±0.6%	0.6 ±0.1%
Bacillus	0.02 ±0.01%	0.5 ±0.3%
Candidatus Saccharimonas	0.5 ±0.4%	0.5 ±0.2%
Bacteroides	0.8 ±0.3%	0.6 ±0.2%
Lachnospiraceae NK4A136 group	0.7 ±0.5%	0.6 ±0.3%
Alloprevotella	0.09 ±0.06%	0.6 ±0.4%
Roseburia	0.5 ±0.2%	0.5 ±0.3%

Table S4. Bacterial genera with low relative abundance (<0.5%).

Bacterial Genera	Goats	Cows
Campylobacter	0.205487039	0.068372032
Unclassified genus within family Bacteroidales RF16 group	0.034904713	0.146632321
Candidatus Soleaferrea	0.090622646	0.118998084
Aeriscardovia	0.19412372	0.009989841
Oscillibacter	0.112159937	0.26544436
Unclassified genus within family F082	0.072324842	0.290483833
Unclassified genus within order Bacteroidales	0.437372695	0.040015672
Acinetobacter	0.094868718	0.007357635
Turicibacter	0.068361282	0.001323213
Olsenella	0.092104969	0.004248563
Unclassified genus within family Clostridiales vadinBB60 group	0.287309216	0.001223652
Unclassified genus within order Clostridiales	0.008928542	0.00289023
Ruminiclostridium	0.044308574	0.002383758
Acetitomaculum	0.111697452	0.371959423
Ruminococcus 2	0.38557856	0.000280199
Unclassified genus within family F082	0.039164039	0
Unclassified genus within order Rhodospirillales	0.393251765	0.392198582
Ruminiclostridium 1	0.196015514	0.009298194
Anaerovorax	0.194184408	0.005645208
Lachnospiraceae UCG-001	0.02399202	0.130025831
Unclassified genus within order Bacillales	0.008109183	0.182411911
Hydrogenoanaerobacterium	0.05441	0.038661984
Ruminiclostridium 5	0.118847485	0.192531491
Unclassified genus within family p-251-o5	0.147796395	0.218738607
Parasutterella	0.083633751	0.249746037
Streptococcus	0.034878205	0.100936321
p-1088-a5 gut group	0.128003013	0.121434956
Tyzzerella 4	0.110738302	0.295803099
Prevotella 1	0.215896091	0.097044175
Negativibacillus	0.026507436	0.216014105
Eubacterium hallii group	0.026333045	0.134797991
Lachnospiraceae FCS020 group	0.299603784	0.062404075
Unclassified genus within family vadinBE97	0.053538045	0.132981656
Unclassified genus within order DTU014	0.038714808	0.063831195
Saccharofermentans	0.054322805	0.077583444
Defluviitaleaceae UCG-011	0.004299088	0.018969931
Unclassified genus within family Mitochondria	0.00252867	0.017283334
Ruminiclostridium 6	0.049003878	0.070188367
Unclassified genus within family Victivallaceae	0.151371411	0.044556509

Victivallis	0.022060465	0.064739362
Prevotellaceae UCG-001	0.054497196	0.002233703
Gordonibacter	0.064175898	0.007135601
Unclassified genus within family Barnesiellaceae	0.119819889	0.254935566
Eubacterium oxidoreducens group	0.008631379	0.051116851
Ruminococcaceae UCG-004	0.002160914	0.150107099
Ruminobacter	0.054845978	0.017644395
Lachnoclostridium	0.088939424	0.047354443
Unclassified genus within order Mollicutes RF39	0.084666843	0.012353282
Desulfovibrio	0.047259968	0
Unclassified genus within family vadinBE97	0.19706186	0.010547716
Odoribacter	0.02371718	0.061755384
Lachnospiraceae UCG-006	0.049701443	0.063312242
Lachnoclostridium 10	0.066007004	0.064350148
Porphyromonas	0.068535673	0
Unclassified genus within family Christensenellaceae	0.11640601	0.06564753
Prevotellaceae Ga6A1 group	0.036883702	0.016087537
Garciella	0	0.063960933
Eubacterium ventriosum group	0.048655096	0.067593603
CHKCI002	0.024240353	0.016476752
Unclassified genus within order Victivallales;__;__	0.017090321	0.008951936
CPla-4 termite group	0.037145289	0
Unclassified genus within order Rhodospirillales	0.036186138	0.029710048
Unclassified genus within family Victivallaceae	0.040458718	0.097822604
Unclassified genus within order Izimaplasmatales	0.035837356	0.001556858
DNF00809	0.098356539	0.081086376
Unclassified genus within family Muribaculaceae	0.043772148	0.031266907
Selenomonas 1	0.023368398	0.030488477
uncultured Parabacteroides sp.	0.020229359	0.084719045
unidentified bacterium RFN82	0.009242724	0.026466593
Unclassified genus within family Eggerthellaceae	0.02894891	0.034380623
Unclassified genus within family Victivallaceae	0.035052596	0.002724502
Unclassified genus within family Eggerthellaceae	0.020926923	0.014660417
Lachnospiraceae UCG-008	0.069233238	0.076415801
Elusimicrobium	0.031651971	0.048651825
Aerococcus	0.009233238	0.006227434
Blautia	0.056241106	0.078751088
Collinsella	0	0.02088785
Unclassified genus within family Corynebacteriaceae	0	0.040867533
Unclassified genus within family Rikenellaceae	0.049788638	0.022574447
Lachnospiraceae UCG-004	0.002092692	0.024131305
Marvinbryantia	0.036186138	0.080437685
Microbacterium	0.006365272	0.033731932

Parabacteroides	0.008196378	0.014660417
Unclassified genus within family Lachnospiraceae	0.002615865	0.030358739
Ruminococcaceae UCG-008	0.009591506	0.02737476
Murdochiella	0.015259215	0
Stenotrophomonas	0.004708558	0.029191095
Anaerococcus	0.035139792	0
Caproiciproducens	0.032175144	0.007524816
Syntrophorhabdus	0.01185859	0
Bilophila	0.015782388	0.007135601
Unclassified genus within order Gastranaerophilales	0.024501939	0.00505979
Lachnospiraceae XPB1014 group	0.014474455	0.005189528
Coprococcus 2	0.023455593	0.018163348
Eubacterium eligens group	0.01107383	0.004800313
Atopobium	0.035488574	0.007654554
Ruminococcus gauvreauii group	0.030780016	0.031656121
Lachnospiraceae NC2004 group	0.016043974	0
Unclassified genus within family Muribaculaceae	0.009765897	0.019849945
Pseudobutyrivibrio	0.02371718	0.005578743
Corynebacterium 1	0.01787508	0.00363267
Unclassified genus within family Clostridiaceae 1	0	0.024131305
Ruminococcaceae UCG-001	0.026594632	0
uncultured Kiritimatiellaeota bacterium	0.010899439	0.005169211
Unclassified genus within order WCHB1-41	0.012294567	0.003742091
Cellulosilyticum	0.006278077	0.003581877
Ruminococcus gnavus group	0.006539663	0.000239159
Unclassified genus within family Planococcaceae	0.006714055	0.019330992
Ruminococcus torques group	0.011335417	0.016476752
Lachnospiraceae UCG-002	0.03400625	0.015698322
Lachnospiraceae NK4B4 group	0.012904936	0
Cutibacterium	0.009678702	0
Unclassified genus within family Clostridiales vadinBB60 group	0.007411619	0.014790155
Howardella	0.014561651	0.00363267
Unclassified genus within family Bacteroidales BS11 gut group	0.008109183	0
Lachnospira	0	0.01946073
Eubacterium xylanophilum group	0.016305561	0.002983979
uncultured Porphyromonadaceae bacterium	0	0.013103558
GCA-900066575	0.016828734	0.021147327
Unclassified genus within family Lachnospiraceae	0.007760401	0
Coprococcus 3	0.02040375	0.010768271
Unclassified genus within family Clostridiales vadinBB60 group	0.007237228	0.004021884
Butyrivibrio	0.009765897	0.014141464

Agathobacter	0.007498814	0.00363267
S5-A14a	0.009765897	0
Family XIII UCG-001	0.019095817	0.000648691
Unclassified genus within family gir-aah93h0	0.000174391	0.013492773
Unclassified genus within order Mollicutes RF39	0.007237228	0.004670575
FD2005	0.007760401	0
Unclassified genus within order Micrococcales	0.006190881	0
Anaerosporobacter	0.004185385	0.001556858
Finegoldia	0.004474455	0
Eubacterium ruminantium group	0.000962276	0.031266907
Unclassified genus within order Rhodospirillales	0.011771394	0.002335288
Papillibacter	0.003836603	0
Barnesiella	0.003749407	0
Enterococcus	0.000539663	0.00142712
Acetobacter	0	0.013233297
Fastidiosipila	0.004446971	0
Peptococcus	0.011248221	0.009211412
Enterorhabdus	0.003400625	0
Negativicoccus	0.00505734	0
Faecalibacterium	0.006975641	0.010898009
Anaerotruncus	0.002877452	0.012195391
Syntrophococcus	0.013340914	0.013752249
Unclassified genus within family Dysgonomonadaceae	0.005231731	0
Unclassified genus within family Actinomycetaceae	0	0.008043768
Unclassified genus within family Ruminococcaceae	0.002964647	0.005578743
Butyrivibrio 2	0	0.009211412
Unclassified genus within family Veillonellaceae	0.001918301	0.015568584
Unclassified genus within order OPB41	0	0.004151622
Peptoniphilus	0.003923798	0
Pseudomonas	0.000610369	0.006357172
Saccharomonospora	0	0.00648691
Devosia	0	0.005967957
Gallicola	0.003836603	0
Sarcina	0	0.007265339
Tyzzerella	0.004098189	0.003892146
Prevotella 7	0.00331343	0
Ruminococcaceae UCG-011	0.002179888	0.000648691
Methylobacterium	0	0.004411099
Curtobacterium	0	0.006746386
Delftia	0	0.004800313
horsej-a03	0	0.004800313
Unclassified genus within order WCHB1-41	0	0.007784292
Lachnospiraceae FE2018 group	0.002615865	0

Allorhizobium-Neorhizobium-Pararhizobium-Rhizobium	0.000348782	0.006357172
Lachnospiraceae UCG-010	0	0.006876125
Mycobacterium	0.000174391	0.00505979
Lactobacillus	0	0.008692459
Unclassified genus within order WCHB1-41	0	0.002724502
Unclassified genus within order Mollicutes RF39	0.003139038	0.006616648
Parvibacter	0.001656715	0
Succiniclasticum	0.002354279	0
Thermobifida	0	0.002983979
Tyzzerella 3	0.000871955	0.001686597
Unclassified genus within family Nocardiaceae	0	0.002205549
Paenibacillus	0.002703061	0
Incertae Sedis	0	0.002075811
Micromonospora	0	0.002075811
Shuttleworthia	0.002615865	0
Libanicoccus	0	0.001686597
Unclassified genus within family Planococcaceae	0	0.001686597
Unclassified genus within family Propionibacteriaceae	0.001046346	0
Oceanobacillus	0	0.001556858
Enhydrobacter	0	0.001556858
Anaerovibrio	0	0.001556858
Sphingobacterium	0	0.002724502
Unclassified genus within family Microbacteriaceae	0	0.002594764
Dolosigranulum	0	0.001297382
Mycoplasma	0	0.002983979
Pediococcus	0	0.002335288
Unclassified genus within order Coriobacteriales	0.000871955	0
SP3-e08	0.00078476	0
Unclassified genus within family Thermoactinomycetaceae	0	0.001167644
Aureimonas	0	0.001167644
UBA1819	0	0.001167644
Eisenbergiella	0.00078476	0
Nocardioides	0	0.001037906
Prevotellaceae YAB2003 group	0.000959151	0.001037906
Unclassified genus within family Dermatophilaceae	0	0.000908167
Schwartzia	0	0.000908167
Succinivibrio	0	0.000778429
Unclassified genus within family vadinBE97	0	0.000778429
Thermoactinomyces	0	0.000778429
Pirellula	0	0.000778429
Sutterella	0.00078476	0
Unclassified genus within family Paracaedibacteraceae	0.000435978	0
Saccharopolyspora	0	0.000648691

Weissella	0	0.000648691
Lawsonella	0.00078476	0
Lachnospiraceae ND3007 group	0.000348782	0
Pelistega	0	0.000518953
Fibrobacter	0	0.000518953
Helicobacter	0	0.000518953
Unclassified genus within family Rhizobiaceae	0.000348782	0
Tumebacillus	0.000261587	0
Butyricimonas	0.000261587	0
Brachybacterium	0.000261587	0
Clostridium sensu stricto 18	0	0.000389215
Candidatus Endomicrobium	0.000261587	0
Brevundimonas	0.000174391	0
Arcanobacterium	0.000174391	0
Lachnoclostridium 12	0	0.000259476
Unclassified genus within family Solirubrobacteraceae	0	0.000259476
Unclassified genus within family Fusobacteriaceae	0.000174391	0

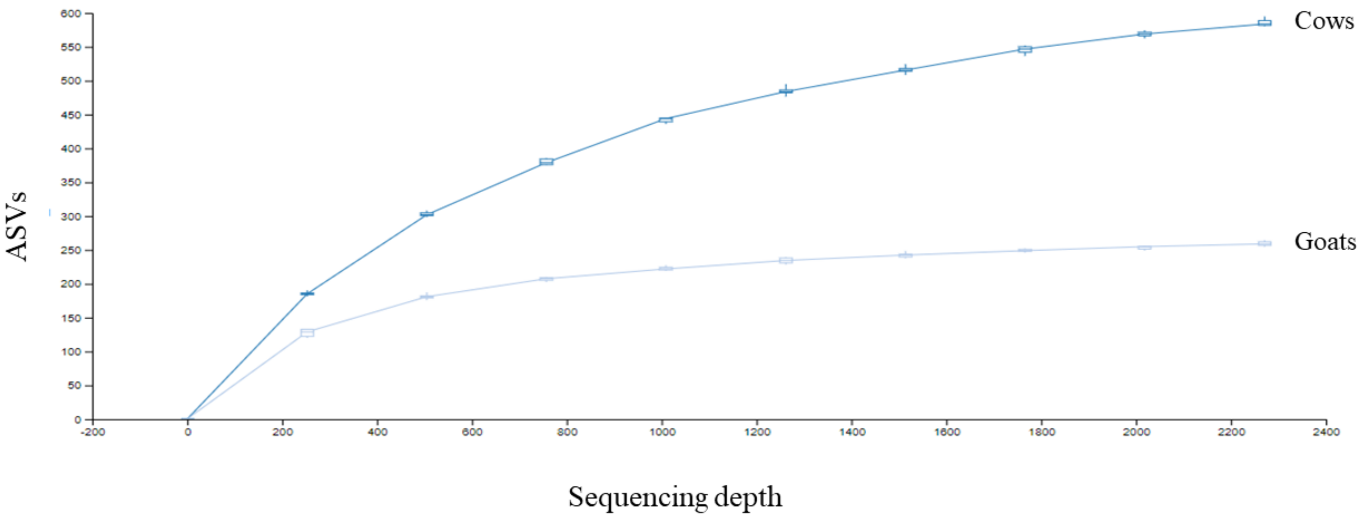

Figure S1. Rarefaction curves representing the sequencing depth (number of reads) and the number of ASVs (sequence variants) found in feces of goats and cows.

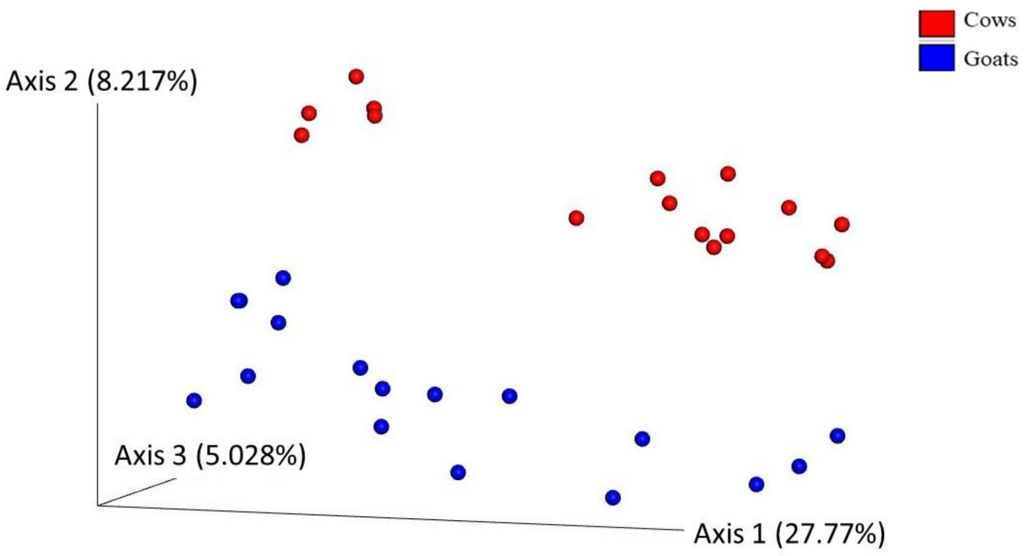

Figure S2. Principal Coordinate Analysis (PCoA) showing the unweighted UniFrac distance matrix of bacterial 16S rRNA amplicons from fecal samples of cow (red color) and goat (blue color) groups. Each dot represents one sample. The percentage of variation explained by the plotted principal coordinates is indicated on the axes.
